# Use of complementary and alternative therapies for the treatment of dysmenorrhea among undergraduate pharmacy students in Malaysia: a cross sectional study

**DOI:** 10.1186/s12906-020-03082-4

**Published:** 2020-09-18

**Authors:** Usman Abubakar, Amni Izzati Zulkarnain, Fatihah Samri, Sabrina Ros Hisham, Anis Alias, Muhammad Ishak, Hajarah Sugiman, Taufik Ghozali

**Affiliations:** Department of Pharmacy Practice, Kulliyah of Pharmacy, International Islamic University Malaysia, 25200 Kuantan, Pahang Malaysia

**Keywords:** Dysmenorrhea, Pharmacy students, Complementary and alternative therapy, Predictors, Perception, Malaysia

## Abstract

**Background:**

Dysmenorrhea is a common problem that affects female students’ quality of life and academic activities. Complementary and alternative therapies (CATs) are used for the treatment of dysmenorrhea. This study investigated the practices and perceptions of female undergraduate students with dysmenorrhea towards CATs.

**Methods:**

This was a cross-sectional study conducted among undergraduate pharmacy students in a public university in Malaysia using a validated and pre-tested self-administered questionnaire. The study was conducted in November and December 2019. The data was analysed using descriptive and inferential statistical tests.

**Results:**

Of the 318 female undergraduate students invited, 219 completed the questionnaire (response rate: 68.9%) with 52% aged between 21 and 23 years. The prevalence of dysmenorrhea was 72.1%, and the prevalence of ever-use and current use of CATs was 70.3 and 54.4%, respectively. Bed rest (71.5%), hot compress/heating pad (47.5%) and massage (43.0%) were the most common CATs used by the respondents. The most common reasons for using CAT were to reduce the need for analgesics (61.4%), efficacy (37.3%) and recommendation by others (32.9%). About 23 and 9% of the respondents believed that CATs were equally “effective” and “more effective” than analgesics, respectively. Reducing the need for analgesics (AOR: 4.066, 95% CI: 2.136–7.739) and those who agreed that CATs are effective (AOR: 2.701, 95% CI: 1.337–5.457) were independently associated with the current use CATs for the treatment of menstrual pain.

**Conclusion:**

The prevalence of ever-use and current use of CATs is high among female undergraduate pharmacy students. Bed rest and heat applications are the most common CATs used. Reducing the need for analgesics and efficacy are the factors associated with the current use of CATs. Students should be educated about the safe and effective use of CATs to reduce adverse effects and improve their quality of life.

## Background

Dysmenorrhea is a public health problem that affects adolescent girls and women of child bearing age [1]. The prevalence of dysmenorrhea ranges between 34 and 94% worldwide with severe dysmenorrhea reported in about 1–60% of the cases [1]. Dysmenorrhea is classified into primary and secondary dysmenorrhea. In primary dysmenorrhea there is no underlying uterine pathology while secondary dysmenorrhea is associated with pelvic pathology [2]. Factors that increase the risk of dysmenorrhea include a positive family history, age, stress, age at menarche, obesity, irregular cycle, long cycle, and heavy bleeding as well as skipping breakfast, eating snacks, smoking and alcohol use [1, 3–5]. The condition causes symptoms such as lower abdominal cramp, low back pain, nausea, vomiting, diarrhoea, headache and fatigue, as well as emotional instability, loss of appetite, sleeplessness and depression [1, 3, 4, 6, 7].

The exact mechanism of dysmenorrhea is unknown, however, studies have suggested that prostaglandins play a major role in the pathogenesis of primary dysmenorrhea [2]. Unfertilized egg (ovum) causes a decrease in progesterone level resulting in the contraction of the uterus and shedding of its lining. The reduction in the level of progesterone leads to an increase in prostaglandin production. Prostaglandin hyper-secretion results in vasoconstriction and myometrial contractions, which cause a reduction in uterine blood flow and pain [2]. Dysmenorrhea has a negative impact on daily physical activities including daily chores; work or school activities such as absenteeism, loss of concentration, and reduced participation or productivity; as well as social interactions and psychological status [1, 4, 7, 8]. Most women and adolescents with dysmenorrhea do not seek medical treatment from healthcare professionals and most of them resort to self-medication with analgesics and complementary and alternative therapies (CATs) [1, 9]. There is a growing interest for the use of CATs among women with reproductive health issues [10]. Previous studies revealed that 15.5–79.3% of women with dysmenorrhea use CATs [11–13]. Self-medication therapies used by most women with dysmenorrhea are not effective in the treatment of menstrual pain [9]. Therefore, counselling on appropriate use of CATs and management of dysmenorrhea are recommended.

In Malaysia, approximately 56–85% of adult women use CATs for women health issues including: pregnancy, labour, post-menopausal symptoms and infertility [14, 15]. The use of CATs among women is associated with both beneficial and adverse health outcomes [14]. There is limited data describing the use of CATs for the treatment of dysmenorrhea among women and young adults in Malaysia. This information is important to understand the usage of CATs for menstrual pain and to design interventions to promote safe and effective use of CATs. The objective of this study was to investigate the practices and perceptions of female undergraduate pharmacy students towards the use of CATs for the treatment of dysmenorrhea in Malaysia.

## Method

### Study design and setting

This study was a cross sectional study conducted among female undergraduate pharmacy students in a public university located in the East Coast of the Malaysian peninsular using a validated and pre-tested questionnaire. The school of pharmacy involved in this study has a population of 474 undergraduate students and females represent 74.3% of the population. The school offers a 4-year Bachelor of Pharmacy degree program.

### Study population and sample size

All female undergraduate students in all four levels (year 1, 2, 3 and 4) in the school of pharmacy were invited to participate in the study. Students who provided consent to participate in the study were included. Those who participated in the pilot study, those with amenorrhea, those who declined to participate and female postgraduate students in the school as well as students from other schools were excluded from the study. There were 474 undergraduate pharmacy students (348 of whom are females) at the school at the time of the survey. The sample size was calculated using Raosoft Sample Size Calculator using the following assumptions; 95% confidence interval, 5% margin of error and 50% response distribution. The minimum sample size required was calculated as 183 female undergraduate pharmacy students. A convenient sampling technique was used to recruit participants in this study.

### Survey instrument

The questionnaire was developed in the English language after the review of relevant previous literatures on dysmenorrhea and the usage of CAT as well as analgesics [16–24]. The initial questionnaire was validated by three senior lecturers in the Department of Pharmacy Practice and the questionnaire was revised based on the comments and suggestions of the reviewers. The final questionnaire (see supplementary material) consists of 38 items divided into 6 sections as follows; sections A and B that collected data on demographic (3 items) and menstrual (6 items) characteristics, respectively; section C assessed menstrual pain, level of severity and its effect on daily and academic activities (11 items). Section D and E gathered information on practices towards analgesics (6 items) and CATs (5 items), respectively. The last section assessed the perception towards the usage of analgesics and complementary and alternative therapy in the management of dysmenorrhea (7 items). In this report, we described the practices and perception towards the use of CATs among female undergraduate students. The results for the prevalence of dysmenorrhea and its effect on academic activities will be published elsewhere. The severity of dysmenorrhea was assessed using WaLIDD scale [20]. WaLIDD score measures the severity of dysmenorrhea on a scale of 0–12 with scores 0, 1–4, 5–7, and 8–12 representing no, mild, moderate and severe dysmenorrhea, respectively [20]. A five-point Likert scale was used to assess the student’s perceptions towards analgesics and CATs. A pilot study was conducted among 30 students in the school to get feedbacks to the questions in the questionnaire. The data was analysed and the Cronbach alpha value was 0.704. In this study, dysmenorrhea is defined as the presence of pelvic, lower abdominal or back pain during menstruation [25]. CATs users were defined as those who used or are currently using traditional Chinese, Indian and Malay medicines, homeopathy, yoga, exercise, massage, cupping, bed rest, hot compresses, supplements and spiritual/prayer for the treatment of dysmenorrhea.

### Data collection

The data was collected in November and December 2019. A self-administered questionnaire was distributed to the students. A cover letter was attached to the questionnaire and it explained the objective of the study to the potential respondents. Participants were informed that participation in the study is voluntary and all respondents were required to provide consent before they complete the questionnaire. The respondents were given the option to either complete the questionnaire on the spot or submit the completed questionnaire later. Reminders were sent to non-respondents every week through the WhatsApp group of the respective student levels.

### Data analysis

The data was entered, double checked for coding errors, and analysed using Statistical Package for the Social Sciences (SPSS) version 23. Categorical data was described as frequency and percentage while continuous data was presented as mean (standard deviation). Associations between demographics and menstrual characteristics, dysmenorrhea as well as perceptions with the current use of complementary and alternative therapies were assessed using univariate and multivariate logistic regression analyses. The variables with *p* <  0.05 in the univariate regression analysis were included in the multivariate regression model. A value of p <  0.05 was considered to be statistically significant.

## Results

### Demographic and menstrual characteristics of the respondents

Of the 318 (348 female students minus 30 who participated in the pilot study) female students invited to participate in the survey, 219 students completed the questionnaire; corresponding to a 68.9% response rate. Fifty two percent of the respondents were aged 21–23 years while the mean age at menarche was 11.9 ± 1.2 years. Most of the respondents (81.3%) had regular menstruation while 50.7% had a family history of dysmenorrhea and 73.1% had normal menstrual flow (Table 1).

**Table 1:** Demographic and menstrual characteristics of the students who participated in the study

Variable	Frequency (***N*** = 219)	Percentage (%)
Age (years)		
Less than 18	1	0.5
18–20	102	46.6
21–23	114	52.1
24 and above	2	0.9
Year of study		
Year 1	61	27.9
Year 2	46	21.0
Year 3	69	31.5
Year 4	43	19.6
Mean age at menarche (SD)	11.9 (1.2)	
Regularity of menses		
Regular	178	81.3
Irregular	41	18.7
Length of menstrual cycle (days)		
Irregular	19	8.7
Less than 20	21	9.6
21–35	170	77.6
More than 35	9	4.1
Duration of menstruation (days)		
Less than 5	1	0.5
5–7	121	55.3
8 and above	96	43.8
Menstrual flow		
Scanty	39	17.8
Normal	160	73.1
Heavy	20	9.1
Family history		
Yes	111	50.7
No	108	49.3

### Prevalence of dysmenorrhea among the respondents

Of the 219 respondents, 72.1% indicated that they experienced menstrual pain (dysmenorrhea) during their menstruation. The symptoms reported by the respondents include cramp in lower abdomen (88.0%), fatigue (52.5%), headache (31.6%) and nausea and vomiting (16.5%). The severity of dysmenorrhea, assessed using WaLIDD scale, showed that 60.7, 24.1 and 15.2% of the respondents had moderate, severe and mild dysmenorrhea, respectively (Table 2).

**Table 2:** Prevalence, symptoms and severity of dysmenorrhea among the students

Variable	Frequency (N = 219)	Percentage (%)
Prevalence of dysmenorrhea	158	72.1
Symptoms of dysmenorrhea		
Cramp in lower abdomen	139	88.0
Pain radiating to the leg	42	26.6
Fatigue	83	52.5
Headache	50	31.6
Diarrhoea	17	10.8
Nausea and vomiting	26	16.5
Fainting	4	2.5
Multiple symptoms (> 3)	111	70.3
Severity of dysmenorrhea^a^		
Mild	24	15.2
Moderate	96	60.7
Severe	38	24.1

^a^Severity of dysmenorrhea was assessed using WaLIDD scale. WaLIDD score = 0, 1–4, 5–7, and 8–12 represented no, mild, moderate and severe dysmenorrhea, respectively; *SD* Standard deviation

### Use of complementary and alternative therapies for the treatment of dysmenorrhea

More than two-third (70.3%) of the respondents had used CATs before while 54.4% were current users of CATs. Bed rest (71.5%), hot compress/heating pad (47.5%) and massage (43.0%) were the most common complementary and alternative therapies used by the students for the treatment of dysmenorrhea. Natural herbs/remedies/supplements (10.1%) and exercise/yoga/meditation (9.5%) were the least common CATs used by the students. The reasons for the usage of CATs among the students included to reduce the need for analgesics (61.4%), efficacy (37.3%) and based on recommendation (32.9%). Availability (17.1%) and cost (6.3%) were the least common reasons for the usage of CATs for the treatment of dysmenorrhea. Approximately 41% of the students with dysmenorrhea used analgesics for the treatment of their pain. One-third of the students believed that CATs are less effective than analgesics, while 22.8 and 8.9% indicated that CATs are equally effective and more effectively than analgesics, respectively (Table 3).

**Table 3:** Prevalence, types and reasons for the use of complementary and alternative therapies among the respondents

Variable	Frequency (***N*** = 158)	Percentage (%)
Ever-user of CAT	111	70.3
Current user of CAT	86	54.4
Types of CATs used for dysmenorrhea		
Bed rest	113	71.5
Hot compress/heating pad	75	47.5
Massage	68	43.0
Hydrotherapy	42	26.6
Natural herbs/remedies/supplements	16	10.1
Exercise/yoga/meditation	15	9.5
Others	6	3.8
Reasons for using CATs		
To reduce the need for analgesics	97	61.4
Efficacy	59	37.3
Based on recommendation	52	32.9
Safety	33	20.9
Availability	27	17.1
Cost	10	6.3
Effectiveness of CATs compared to analgesics		
Less effective	47	29.7
Equally effective	36	22.8
More effective	14	8.9
I don’t know	60	38.0
Use of analgesic for dysmenorrhea	64	40.5

*CAT* Complementary and alternative therapy.

### Perceptions towards the use of complementary and alternative therapies for dysmenorrhea

Approximately 61 and 12.0% of the students agreed and strongly agreed, respectively, that CATs were effective in relieving menstrual pain. About 50% neither agreed nor disagreed that CATs are safer than analgesics for relieving menstrual pain. Only 15.2 and 33.5% strongly agreed and agreed, respectively, that CATs are safer than analgesics (Table 4).

**Table 4:** Respondent’s perception towards complementary and alternative therapies

Variable	Frequency (%)				
	Strongly agree	Agree	Neutral	Disagree	Strongly disagree
CATs are effective in relieving menstrual pain	19 (12.0)	96 (60.8)	43 (27.2)	0 (0.0)	0 (0.0)
CATs are safer than analgesics in relieving menstrual pain	24 (15.2)	53 (33.5)	78 (49.4)	3 (1.9)	0 (0.0)

*CAT* Complementary and alternative therapy.

### Factors associated with the current use of complementary and alternative therapies

Univariate logistic regression analysis showed that students with multiple (three or more) symptoms of dysmenorrhea (OR: 2.377, 95% confidence interval (CI): 1.366–4.136, *P* = 0.002), those who use analgesics (OR: 2.317, 95% CI: 1.369–4.129, *P* = 0.004), those who want to reduce the need for analgesics (OR: 5.557, 95% CI: 3.099–9.967, *P* <  0.001) and those who agreed that CATs are effective for the treatment of dysmenorrhea (OR: 3.233, 95% CI: 1.740–6.007, *P* <  0.001) were significantly associated with the current use of CATs for the treatment of dysmenorrhea. There was no significant association between demographic and other menstrual characteristics with the current use of CATs. Multivariate logistic regression analysis demonstrated that those who want to reduce the need for analgesics (AOR: 4.066, 95% CI: 2.136–7.739) and those who agreed that CATs are effective for the treatment of dysmenorrhea (AOR: 2.701, 95% CI: 1.337–5.457) were independently associated with the current use CATs for the treatment of dysmenorrhea (Table 5).

**Table 5:** Univariate and multivariate regression analyses of factors associated with the current use of complementary and alternative therapies among the students

Variable	Univariate logistic regression analysis			Multivariate logistic regression analysis		
	Odds ratio	95% CI	***P*** value	Adjusted Odds ratio	95% CI	P value
Those who have multiple dysmenorrhea symptoms	2.377	1.366–4.136	**0.002**	1.160	0.565–2.382	0.687
Those who use analgesics	2.317	1.300–4.129	**0.004**	1.369	0.664–2.821	0.395
WaLIDD score (continuous variable)	1.274	1.109–1.463	**0.001**	1.102	0.913–1.329	0.312
Those who want to reduce the need for analgesics	5.557	3.099–9.967	**< 0.001**	4.066	2.136–7.739	**< 0.001**
Those who agreed that CATs are effective in relieving menstrual pain	3.233	1.740–6.007	**< 0.001**	2.701	1.337–5.457	**0.006**
Those who agreed that CATs are safer than analgesics in relieving menstrual pain	2.577	1.494–4.447	**0.001**	1.703	0.910–3.185	0.096

## Discussion

The current study found that more than two-third of female undergraduate pharmacy students experienced dysmenorrhea and this result corroborate previous findings in Malaysia 62–72% [26, 27]. It was also found that among those with dysmenorrhea, about two-third had moderate dysmenorrhea and this was higher than previous studies conducted in Turkey and Mexico, where moderate dysmenorrhea accounts for less than 50% of the cases [21, 28], although different scales were used to classify the severity of the symptoms. Clinical evidence demonstrates that CATs including exercise, yoga, heat, acupressure and home remedies such as oral ginger are effective in alleviating menstrual pain in women with dysmenorrhea [29–31]. The current study revealed that more than two-third and a little more than half of the students had used and were current users of CATs for the treatment of dysmenorrhea, respectively. This was not consistent with the result of a similar study conducted among high school girls in Ghana [12] and could be attributed to the differences in age, educational background and socioeconomic status of the respondents. Our result is consistent with the result of an Australian study which revealed high rate of CATs use among women with cyclic peri-menstrual pain and discomfort [32].

The most common CAT used by the students was bed rest, and this corroborates the finding of a Ghanaian study [4]. This is because sleep may be considered as a positive distraction from dysmenorrhea-associated pain. Heat application has been found to have a moderate effect in alleviating pain associated with dysmenorrhea and it is considered as an alternative to analgesics [31, 33]. This is because heat application improves blood flow and causes relaxation of the uterine muscles which results in reduction in pain [10, 31]. The current study found that hot compress/heating pad was the second most common CAT used by the students to relieve menstrual pain and this was not in agreement with the result of a previous study [4]. Natural herbs/remedies/supplements and exercise/yoga/meditation were the least common CATs used for menstrual pain, similar with a previous study [4]. The reason for this observation is unknown. Future studies should investigate the reasons for the low usage of natural herbs and remedies in the treatment of dysmenorrhea.

The current study also revealed that most of the students used CATs for the treatment of dysmenorrhea in order to reduce the need for analgesics. This could be attributed to the fear of the side effects associated with analgesics, particularly non-steroidal anti-inflammatory drugs (NSAIDs) which cause gastrointestinal ulcer and chronic kidney disease. In addition, women with dysmenorrhea are reluctant to use analgesic medications due to fears of reduction in menstrual flow and infertility [16]. Also, most of the students used CAT for dysmenorrhea due to its efficacy and safety. The efficacy of CATs have been affirmed in previous studies [29–31, 34], however, there is paucity of data to describe their safety profile. In addition, availability and cost are less common reasons for the use of CATs and this indicate that those who have dysmenorrhea are more interested in relieving their menstrual pain than the cost or availability of the treatment.

Another finding of this study is that about two out of five students with dysmenorrhea used analgesics to relieve pain. This was lower than the rate of analgesic use for dysmenorrhea in previous studies conducted in Turkey and Saudi Arabia [6, 19]. The difference could be explained by the educational background of the students in the current study. Pharmacy students are more likely to be aware of the side effects of analgesics than non-medical based students. Studies have demonstrated that CATs are equally effective compared to analgesics for the treatment of dysmenorrhea [35, 36]. In the current study, less than one-third of the respondents believed that CATs are equally or more effective than analgesics for the treatment of menstrual pain. There was doubt regarding the safety profile of analgesics as one-fifth of the students used CAT due to its safety while approximately 50% strongly agreed/agreed that CATs are safer than analgesics for alleviating menstrual pain. The comparative safety advantage of some CATs including home remedies such as ginger has been previously reported [35], although there is insufficient evidence to support the hypothesis that CATs are associated with fewer side effects than analgesics.

The present study found that there was no association between the current use of CATs and menstrual characteristics. However, the number of symptoms (multiple) and the severity of the symptoms (WaLIDD score) were significantly associated with current use of CATs for dysmenorrhea. These reflect the desire to relieve menstrual pain among those with severe dysmenorrhea because they have more difficulty with daily chores and academic activities compared to those with mild dysmenorrhea [7]. There was also significant association between the need to reduce analgesic use and the current use of CATs suggesting the possibility of using both therapies concurrently and highlights to need for education to promote appropriate health seeking behaviours for the treatment of dysmenorrhea. The current study also revealed that the efficacy of CATs and those who want to reduce the need for analgesics were independently associated with the current use of CATs. The high rate of CATs use among women and adolescents with dysmenorrhea warrants further research to ascertain their effectiveness and safety in the management of dysmenorrhea. There are weak evidence to support the use of CATs in dysmenorrhea [10], and their safety profile have not been adequately investigated [31]. Healthcare providers and community pharmacists should ask women and adolescents with dysmenorrhea about the use of CATs and provide counselling on the safe and effectively use of CATs and conventional medicines considering the potential for interaction between both medicines.

This study has some limitations and thus, the result should be interpreted with caution. First, the study was conducted among undergraduate students in one pharmacy school and thus the results may not be generalizable. Secondly, the study did not distinguish between primary and secondary dysmenorrhea and this could affect the result of the study considering the fact that there are different treatment approaches for the two conditions. Thirdly, the study required respondents to recall historical information and could be susceptible to recall bias. Fourthly, there responses provided by the students were self-reported and could be influenced by social desirability bias. Fifthly, the current study used a cross-sectional design to determine causal association between variables as against longitudinal study design which is more reliable. Lastly, dysmenorrhea is a sensitive issue among women and this may have had an influence on the response rate. Despite these limitations, this study has provided information about the prevalence and factors associated with the use of CATs for the treatment of dysmenorrhea among university students. Future studies should target a larger population and distinguish between primary and secondary dysmenorrhea as well as address the limitations described in this study.

## Conclusion

Two-third and a little more than half of pharmacy students with dysmenorrhea are ever- and current users of CATs, respectively. Bed rest, hot compress/heating pad and massage are the most common CATs used by the students. The need to reduce the use of analgesics and believe that CAT is effective in relieving menstrual pain are independently associated with the current use of CATs. Healthcare providers should enquire about the use of CATs among patients with dysmenorrhea and ensure safe and effective use of CATs and conventional medicines.

## Supplementary information

**Additional file 1.**

## Data Availability

The datasets used and/or analysed during the current study are available from the corresponding author on reasonable request.

## Abbreviations

CATs: Complementary and alternative therapies
OR: Odds ratio
AOR: Adjusted odds ratio
CI: Confidence interval
WaLIDD: Working ability, Location, Intensity, Days of pain and Dysmenorrhea
NSAIDs: Non-steroidal anti-inflammatory drugs

## References

[1] De Sanctis V, Soliman AT, Elsedfy H, Soliman NA, Elalaily R, El Kholy M (2016). Dysmenorrhea in adolescents and young adults: a review in different countries. Acta Biomed.

[2] Iacovides S, Avidon I, Baker FC (2015). What we know about primary dysmenorrhea today: a critical review. Hum Reprod Update.

[3] Helwa HA, Mitaeb AA, Al-Hamshri S, Sweileh WM (2018). Prevalence of dysmenorrhea and predictors of its pain intensity among Palestinian female university students. BMC Womens Health.

[4] Ameade EP, Amalba A, Mohammed BS (2018). Prevalence of dysmenorrhea among university students in northern Ghana; its impact and management strategies. BMC Womens Health.

[5] Najafi N, Khalkhali H, Tabrizi FM, Zarrin R (2018). Major dietary patterns in relation to menstrual pain: a nested case control study. BMC Womens Health.

[6] Alsaleem MA (2018). Dysmenorrhea, associated symptoms, and management among students at King Khalid University, Saudi Arabia: an exploratory study. J Family Med Prim Care.

[7] Banikarim C, Chacko MR, Kelder SH (2000). Prevalence and impact of dysmenorrhea on Hispanic female adolescents. Arch Pediatr Adolesc Med.

[8] Schoep ME, Adang EM, Maas JW, De Bie B, Aarts JW, Nieboer TE (2019). Productivity loss due to menstruation-related symptoms: a nationwide cross-sectional survey among 32 748 women. BMJ Open.

[9] Armour M, Parry K, Al-Dabbas MA, Curry C, Holmes K, MacMillan F (2019). Self-care strategies and sources of knowledge on menstruation in 12,526 young women with dysmenorrhea: a systematic review and meta-analysis. PLoS One.

[10] Yu A (2014). Complementary and alternative treatments for primary dysmenorrhea in adolescents. Nurse Pract.

[11] Gebeyehu MB, Mekuria AB, Tefera YG, Andarge DA, Debay YB, Bejiga GS (2017). Prevalence, impact, and management practice of dysmenorrhea among University of Gondar Students, northwestern Ethiopia: a cross-sectional study. Int J Reprod Med.

[12] Samba Conney C, Akwo Kretchy I, Asiedu-Danso M, Allotey-Babington GL (2019). Complementary and alternative medicine use for primary dysmenorrhea among senior high school students in the Western region of Ghana. Obstet Gynecol Int.

[13] Yesuf TA, Eshete NA, Sisay EA (2018). Dysmenorrhea among university health science students, northern Ethiopia: impact and associated factors. Int J Reprod Med.

[14] Yusof J, Mahdy ZA, Noor RM (2016). Use of complementary and alternative medicine in pregnancy and its impact on obstetric outcome. Complement Ther Clin Pract.

[15] Mohamad TA, Islahudin F, Jasamai M, Jamal JA (2019). Preference, perception and predictors of herbal medicine use among Malay women in Malaysia. Patient Prefer Adherence.

[16] Kartal YA (2019). Complementary and alternative medicine therapy use of Western Turkish students for menstrual symptoms. Int J Caring Sci.

[17] Parker MA, Sneddon AE, Arbon P (2010). The menstrual disorder of teenagers (MDOT) study: determining typical menstrual patterns and menstrual disturbance in a large population-based study of Australian teenagers. BJOG.

[18] Kural M, Noor NN, Pandit D, Joshi T, Patil A (2015). Menstrual characteristics and prevalence of dysmenorrhea in college going girls. J Family Med Prim Care.

[19] Oksuz E, Sozen F, Kavas E, Arik EP, Akgun Y, Bingol P (2017). Usage of analgesics among young girls and dysmenorrhea. Konuralp Tıp Dergisi.

[20] Teherán AA, Piñeros LG, Pulido F, Guatibonza MC (2018). WalIDD score, a new tool to diagnose dysmenorrhea and predict medical leave in university students. Int J Womens Health.

[21] Potur DC, Bilgin NC, Komurcu N (2014). Prevalence of dysmenorrhea in university students in Turkey: effect on daily activities and evaluation of different pain management methods. Pain Manag Nurs.

[22] Al-Jefout M, Seham AF, Jameel H, Randa AQ, Luscombe G (2015). Dysmenorrhea: prevalence and impact on quality of life among young adult Jordanian females. J Pediatr Adolesc Gynecol.

[23] Aktaş D (2015). Prevalence and factors affecting dysmenorrhea in female university students: effect on general comfort level. Pain Manag Nurs.

[24] Fernández-Martínez E, Onieva-Zafra MD, Parra-Fernández ML (2018). Lifestyle and prevalence of dysmenorrhea among Spanish female university students. PLoS One.

[25] Osayande AS, Mehulic S (2014). Diagnosis and initial management of dysmenorrhea. Am Fam Physician.

[26] Sukalingam K, Ganesan K (2016). Health-related quality of life in young adult girls with dysmenorrhea among university medical students in Shah Alam, Malaysia: a cross-sectional study. Recent Adv Biol Med.

[27] Jaiprakash H, Myint KK, Chai LY, Nasir BM (2016). Prevalence of dysmenorrhea and its sequel among medical students in a Malaysian University. J Adv Med Medical Res.

[28] Ortiz MI (2010). Primary dysmenorrhea among Mexican university students: prevalence, impact and treatment. Eur J Obstet Gynecol Reprod Biol.

[29] Kim SD (2019). Yoga for menstrual pain in primary dysmenorrhea: a meta-analysis of randomized controlled trials. Complement Ther Clin Pract.

[30] Chen CX, Barrett B, Kwekkeboom KL (2016). Efficacy of oral ginger (zingiber officinale) for dysmenorrhea: a systematic review and meta-analysis. Evid Based Complement Alternat Med.

[31] Armour M, Smith CA, Steel KA, Macmillan F (2019). The effectiveness of self-care and lifestyle interventions in primary dysmenorrhea: a systematic review and meta-analysis. BMC Complement Altern Med.

[32] Fisher C, Adams J, Hickman L, Sibbritt D (2016). The use of complementary and alternative medicine by 7427 Australian women with cyclic perimenstrual pain and discomfort: a cross-sectional study. BMC Complement Altern Med.

[33] Jo J, Lee SH (2018). Heat therapy for primary dysmenorrhea: a systematic review and meta-analysis of its effects on pain relief and quality of life. Sci Rep.

[34] Shah M, Monga A, Patel S, Shah M, Bakshi H (2014). The effect of hypnosis on dysmenorrhea. Int J Clin Exp Hypn.

[35] Shirvani MA, Motahari-Tabari N, Alipour A (2015). The effect of mefenamic acid and ginger on pain relief in primary dysmenorrhea: a randomized clinical trial. Arch Gynecol Obstet.

[36] Salmalian H, Saghebi R, Moghadamnia AA, Bijani A, Faramarzi M, Amiri FN (2014). Comparative effect of thymus vulgaris and ibuprofen on primary dysmenorrhea: a triple-blind clinical study. Caspian J Intern Med.

