# Supplementary material for: Use of complementary and alternative therapies for the treatment of dysmenorrhea among undergraduate pharmacy students in Malaysia: a cross sectional study
Source: BMC Complement Med Ther. 2020 Sep 18;20:285. doi: 10.1186/s12906-020-03082-4 (PMC7501717; doi:10.1186/s12906-020-03082-4)
Supplement: Supplementary file 1 — Additional file 1. [file 12906_2020_3082_MOESM1_ESM.docx]

**Use of analgesics and complementary and alternative therapies for the treatment of menstrual pain among female undergraduate students**

**Questionnaire**

**Section A: Socio-demographic Characteristics**

1. Age:

< 18 years old 8-20 years old 21-23 years old > 24 years old

1. Year of study

Year 1 Year 2 Year 3 Year 4

1. Marital status

Single Married

**Section B: Menstrual characteristics**

1. At what age did you start to menstruate? (Please state:______________)
2. Regularity of menses in the last 12 months?

Regular Irregular

1. How long is your menstrual cycle (in days)?

≤ 21 22 – 28 ≥ 29 days

1. How long is your menstrual period?

< 5 days 5 – 7 days > 8 days

1. Amount of menstrual flow?

Scanty (1 to 2 pads a day) Normal flow (2 to 3 pads a day)

Very heavy (More than 4 pads per day and/or associated with clots)

1. Family history of menstrual pain?

Yes No I don’t know

**Section C: Assessment of menstrual pain and its impact on academic and daily activities**

1. In your last three menstruations, did you experienced menstrual pain?

Yes No I don’t know

1. How often do you experience menstrual pain?

Every menstruation At intervals Rarely Never

1. When do you experience menstrual pain?

Before menses starts During menstruation After menstrual period

All of the above

1. Which of the following symptoms do you experience during menstruation? (You can tick more than one)

Cramping in the lower abdomen Nausea or vomiting Fainting Diarrhoea Headache Fatigue

1. Pain score WaLIDD scale)

i. Does menstrual pain affect your working ability?

None Almost never Almost always Always

ii. Number of body parts experiencing the pain

0 site 1 site 2 - 3 sites 4 sites

iii. Intensity of the pain

Does not hurt Hurts a little bit Hurts a little more - hurts even more

Hurts a whole lot – hurts worse

iv. Days of pain

0 1 – 2 3 – 4 > 4

1. Which of the following daily activities does menstrual pain interfere with? (You may tick more than one)

Daily chores Sleep disturbance Participation in sports

Socializing with peers Meal routine

1. How does menstrual pain affect your academic activities? (You may tick more than one)

Missing class Loss of concentration during class

Lack/reduced participation in class activities Reduced academic performance

1. On an average scale, how many class(es) do you miss when you experience menstrual pain?

I don’t miss class 1 – 3 classes 4 – 5 classes More than 5 classes

**Section D: Usage of analgesics for menstrual pain**

1. Do you take analgesics to relieve menstrual pain?

Yes No I don’t know

1. What kind of analgesic do you take for menstrual pain? (You may tick more than one)

Paracetamol Naproxen Ibuprofen Mefenamic acid

Tramadol Others (please indicate): ……………………

1. What is the main reason for your choice of analgesics in relieving menstrual pain? (You may tick more than one)

Safety Effectiveness Price Availability

Recommendation from others

1. When do you usually take analgesic for menstrual pain?

Before the pain starts At the onset of menstrual pain Upon pain intensification

1. How do you become aware of analgesic use for menstrual pain? (You may tick more than one)

Family/friends Physician/pharmacist Teachers/Lecturers

Social media Television/Radio/Newspaper

1. Do you consult healthcare professionals before taking analgesics for menstrual pain?

Yes No I don’t know

**Section E: Usage of complementary and alternative therapy for menstrual pain**

1. Have you ever used complementary and alternative therapy for menstrual pain?

Yes No I don’t know

1. Are you currently using complementary and alternative therapy for menstrual pain?

Yes No I don’t know

1. What kind of complementary and alternative therapy do you use for menstrual pain? (You may tick more than one)

Natural herbs/remedies/supplements Exercise/yoga/meditation

Hydrotherapy (Drinking a lot of water) Use hot compresses/heating pads

Sleep through the pain (bed rest) Massage Others (indicate): ……..

1. What is the main reason for your choice of complementary and alternative therapy for relieving menstrual pain? (You may tick more than one)

Safety Efficacy To reduce the need for analgesics

Price Recommendation from others Availability

**Section F: Assessment of Perception**

1. Do you believe that analgesics are effective for relieving menstrual pain?

Strongly Agree Agree Neutral Disagree Strongly Disagree

1. Do you think that complementary and alternative are effective for relieving menstrual pain?

Strongly Agree Agree Neutral Disagree Strongly Disagree

1. Do you agree that complementary and alternative therapies are safer than analgesics in relieving menstrual pain?

Strongly Agree Agree Neutral Disagree Strongly Disagree

1. Kindly rate the effectiveness of complementary and alternative therapy compared to analgesic in relieving menstrual pain?

Less effective than analgesic Equally effective as analgesic

More effective than analgesic I don’t know

1. Do you think that long-term consumption of analgesics can cause stomach ulcer?

Strongly Agree Agree Neutral Disagree Strongly Disagree

1. Do you think that long-term consumption of analgesics can cause infertility?

Strongly Agree Agree Neutral Disagree Strongly Disagree

1. Do you think that long-term consumption of analgesics can cause damage to the kidney?

Strongly Agree Agree Neutral Disagree Strongly Disagree
